# Exploration the Mechanism of Doxorubicin-Induced Heart Failure in Rats by Integration of Proteomics and Metabolomics Data

**DOI:** 10.3389/fphar.2020.600561

**Published:** 2020-12-10

**Authors:** Yu Yuan, Simiao Fan, Lexin Shu, Wei Huang, Lijuan Xie, Chenghao Bi, Hongxin Yu, Yuming Wang, Yubo Li

**Affiliations:** Department of Traditional Chinese Medicine, Tianjin University of Traditional Chinese Medicine, Tianjin, China

**Keywords:** doxorubicin, heart failure, proteomics, metabonomics, protein tyrosine-protein phosphatase non-receptor type 1

## Abstract

Heart failure is a common systemic disease with high morbidity and mortality worldwide. Doxorubicin (DOX) is a commonly used anthracycline broad-spectrum antitumor antibiotic with strong antitumor effect and definite curative effect. However, cardiotoxicity is the adverse reaction of drug dose cumulative toxicity, but the mechanism is still unclear. In this study, proteomics and metabonomics techniques were used to analyze the tissue and plasma of DOX-induced heart failure (HF) in rats and to clarify the molecular mechanism of the harmful effects of DOX on cardiac metabolism and function in rats from a new point of view. The results showed that a total of 278 proteins with significant changes were identified by quantitative proteomic analysis, of which 118 proteins were significantly upregulated and 160 proteins were significantly downregulated in myocardial tissue. In the metabonomic analysis, 21 biomarkers such as L-octanoylcarnitine, alpha-ketoglutarate, glutamine, creatine, and sphingosine were detected. Correlation analysis showed that DOX-induced HF mainly affected phenylalanine, tyrosine, and tryptophan biosynthesis, D-glutamine and D-glutamate metabolism, phenylalanine metabolism, biosynthesis of unsaturated fatty acids, and other metabolic pathways, suggesting abnormal amino acid metabolism, fatty acid metabolism, and glycerol phospholipid metabolism. It is worth noting that we have found the key upstream target of DOX-induced HF, PTP1B, which inhibits the expression of HIF-1α by inhibiting the phosphorylation of IRS, leading to disorders of fatty acid metabolism and glycolysis, which together with the decrease of Nrf2, SOD, Cytc, and AK4 proteins lead to oxidative stress. Therefore, we think that PTP1B may play an important role in the development of heart failure induced by doxorubicin and can be used as a potential target for the treatment of heart failure.

## 1 Introduction

Heart disease has a high mortality and morbidity worldwide. Clinically, anthracyclines are a major reason of cardiotoxicity (Feijen et al., 2019). For example, doxorubicin (DOX) is an effective anticancer agent for the treatment of solid tumors and hematological malignancies, which can cause pathological and physiological damage to the heart, such as progressive cardiac dilation and systolic dysfunction, and subsequently lead to heart failure (HF) (Eisenberg et al., 2018; Russo et al., 2019; Maurer and Packer, 2020). Studies have shown that the occurrence of HF caused by DOX is related to cardiomyocyte death caused by mitochondrial swelling, myocardial fibrosis, or cardiac contraction/diastole (Zhang K. W. et al., 2018; Galán-Arriola, 2019). Rochette et al. showed in the study that DOX caused a significant reduction in left ventricular ejection fraction, leading to congestive HF (Rochette et al., 2015). In the treatment of breast cancer patients, HF is the main cause of death due to the use of anthracycline drugs, which can be manifested as, under the condition of no abnormal load, left ventricular dilation and poor function lead to cardiac systolic dysfunction (Zhang et al., 2020). Based on the above studies, when anthracycline is used as a chemotherapeutic agent, cardiac toxicity has become the main limiting factor, and the discovery, prevention, and treatment of it will be an important issue.

In medicine, proteomics and metabonomics are used to help identify patients at risk of disease, detect and identify various molecules at the level of proteins and metabolites, and study their functions and interrelationships among various molecules (Mato et al., 2014). The combined analysis of the two techniques can link genotype and phenotype to help determine the causal mechanism of DOX-induced HF and provide a new strategy for the discovery and effective treatment of HF (Hoffman et al., 2017).

Here, we used ultra-high performance liquid chromatography-tandem quadrupole time-of-flight mass spectrometry (UPLC-Q-TOF/MS) to analyze DOX-induced HF in rats, to explain the toxicological pathways of DOX to the heart, especially the combined proteomic technology, to provide more information for the study of its pathogenesis. We also studied the potential upstream targets for the development of HF.

## 2 Materials and Methods

### 2.1 Chemicals

Doxorubicin (Solarbio, Beijing, China), formaldehyde (Solarbio, Beijing, China), heparin sodium (Solarbio, Beijing, China), acetonitrile (Merck, Germany), formic acid (CNW, Germany), BCA Kit (Solarbio, Shangahi, China), Tris (Sigma, USA), SDS (Bio-Rad, USA), phenylmethylsulfonyl fluoride (Solarbio, Beijing), phosphatase inhibitor (Solarbio, Beijing, China), RIPA buffer (Solarbio, Beijing, China), PTP1B (Abcam, USA), IRS-1, P-IRS, HK2, HIF-1α (Cell Signaling Technology, Inc., USA), Nrf2 (Abcam, USA), NH4HCO3 (Sigma, USA), High pH Reversed-Phase Peptide Fractionation Kit (Pierce, USA), TMT 6/10 plex Isobaric Label Reagent (Thermo, USA) were used.

### 2.2 Animals

A total of 35 male Wistar rats (180–200 g) were purchased from Beijing Weitong Lihua Experimental Animal Technology Co., Ltd., license number: SCXK (Beijing) 2016–0006. After one week of adaptive feeding, the rats were acclimatized to a 12-hour light/dark cycle in a controlled environment with a temperature of approximately 23 ± 2°C and a relative humidity of 35 ± 5%. All animals received care and raising with standard food and tap water. The rats were randomly divided into a treatment group (Dox group) and a control group (NS group). The Dox group was injected intraperitoneally with DOX (3 mg/kg) once a week, and the NS group was injected with the same amount of normal saline intraperitoneally, for 6 weeks.

### 2.3 Compliance With Ethical Standards

This study was approved by the Institutional Animal Care and Use Committee of Tianjin University of Traditional Chinese Medicine (IACUC) and was conducted in accordance with the guidelines of the National Institutes of Health Animal Care and Use Committee.

### 2.4 Biochemical and Pathological Observation

After induction of anesthesia with 5% chloral hydrate, the long-axis parasternal sections of rats were measured using an ultrasound Doppler instrument, and cardiac function indexes were recorded. Ejection fraction (EF) was used as a parameter to determine the establishment of HF. From each serum sample, 100 μL was extracted and measured in a fully automated biochemical instrument, the data of lactate dehydrogenase (LDH), troponin kinase (CK), and creatine kinase isoenzymes (CK–MB) were recorded.

Small sections of heart tissues of NS group and DOX group were fixed with 10% formaldehyde, embedded in paraffin, and then prepared into 5 μm–thick sections. Hematoxylin and eosin (H&E) staining was performed in heart sections to visualize the pathological manifestations under the microscope.

### 2.5 Metabolomics Analysis

We analyzed the plasma of rats in NS group and DOX group by metabonomics. Plasma samples of rats were centrifuged at 4°C and 3500 rpm for 10 min. Each sample was fed with 100 µL of plasma, added to 300 µL acetonitrile (1:3 v/v), and vortexed for 1 min, and then put in ultrasonic ice water bath for 10 min and in the freezing centrifuge (ALLLEGRATM–64R; American) in 13,000 rmp, 4°C/15 min, retaining supernatant. QC samples were prepared to contain biological information of all samples.

To further assess the alterations of metabolites in rats treated with DOX, endogenous metabolites were analyzed by Waters Xevo G2-S UPLC-Q-TOF/MS (Waters, USA). 10 μL of sample solution was injected onto an ACQUITY UPLC BEH C18 (2.1 mm × 100 mm, 1.7 μm, Waters) at 45°C and the flow rate was 0.3 ml/min. The optimal mobile phase consisted of a linear gradient system of a) 0.1% formic acid in water and b) 0.1% formic acid in acetonitrile, 0–8.5 min, 1–25% B; 8.5–11.0 min, 25–50% B; 11.0–13.0 min, 50–1% B; 13.0–15.0 min, 1–1% B. In addition, the QC samples contained most information of whole plasma samples.

The mass spectrum optimal conditions of analysis were as follows: Electrospray ionization source was used, in positive and negative ionization modes, the capillary voltage was 3.0 kV, drying gas temperature was set at 325°C, drying gas flow was 10 ml/min, desolvation gas flow was 600 L/h, source temperature was 120°C, desolvation temperature was 350°C, and cone gas flow was 50 L/h. The quadrupole scan range was set at m/z 50–1000 Da.

### 2.6 Quantitative Proteomics Analysis

Rat myocardial tissue samples were frozen with liquid nitrogen, ground, and crushed with SDT lysate (4% SDS, 100 mM Tris-HCl, 1 mM DTT, pH 7.6) in MP homogenizer. Protein quantification was performed by BCA method. Each sample was 30 ml protein solution, enzymatically hydrolyzed by filter aided proteome preparation (FASP) method (Marwick, 2015), and the peptide segment was quantified under OD280. Each sample was labeled according to the instructions of Thermo TMT labeling kit. The labeled samples were combined and subjected to fractionation (See Supplementary 1 for detailed treatment).

Then each fraction was injected for HPLC analysis, followed by LC–MS/MS analysis performed on a Q Exactive mass spectrometer (Thermo Scientific) that was coupled to Easy nLC (Thermo Fisher Scientific). Buffer A is 0.1% formic acid buffer; B is 84% acetonitrile and 0.1% formic acid aqueous solution. The chromatographic columns used were packed with nanoViper C18 (Thermo Scientific Acclaim PepMap100, 100 μm × 2 cm, 3 μm, 100 A) balanced by 95% A solution at a flow rate of 300 nL/min. The peptides were eluted using a linear gradient (0–80 min, 0–55% B; 80–85 min, 55–100% B; 85–90 min, 100% B).

The detection mode is positive ion, the scanning range of parent ion is 300–1800 m/z, the resolution of primary mass spectrometry is 70,000 at 200 m/z, the target of Automatic Gain Control is 1e6, Maximum IT is 50 m s, and the Dynamic Exclusion time is 60.0 s. The mass–charge ratio of peptides and peptide fragments is collected according to the following methods: 20 fragment spectra (MS2 scan) were collected after each full scan, MS2 Activation Type was HCD, isolation window was 2 m/z, and secondary mass spectrometry resolution was 17,500 at 200 m/z (TMT 6–plex) or 35,000 at 200 m/z (TMT 10–plex), 30 eV for Normalized Collision Energy and 0.1% for Underfill.

### 2.7 Data Processing

MassLynx software (Waters, Version 4.1) was used to extract peak values and correct the original mass spectra data of metabonomics for principal component analysis (PCA) and partial least squares–discriminant analysis (PLS–DA). The standards were variable importance in the projection (VIP) > 1.5 and T-test (*p* < 0.05) to select potential biomarkers. Subsequently according to the HMDB database (http://www.hmdb.ca), the differential metabolites were further identified by m/z values and Mass Spectrometry Fragmentation. The identified metabolites were analyzed by receiver operating characteristic curve and clustering analysis software was used according to the relative content of each biomarker (http://www.metaboanalyst.ca/) to generate a heat map to evaluate the diagnostic ability of metabolic markers. Pathway and visualization analysis was done using the KEGG pathway database (http://mirror.MetaboAnalyst.ca/).

For the tandem mass tag (TMT) proteomic data, MS/MS spectra were searched using MASCOT engine (Matrix Science, London, UK, version 2.2) embedded into Proteome Discoverer 1.4 (Supplementary 1). LC/MS raw data were searched in the UniProt-reviewed rat protein database and differentially expressed proteins were screened by the criteria of upregulation greater than 1.2 times or downregulation less than 0.83 and *p* value less than 0.05. All identified proteins were annotated with Blast2GO for GO function (Database version: go_201504.obo, www.geneontology.org), and then differentially expressed proteins were analyzed by Fisher’s exact test for GO functional enrichment and bioinformatics. Next, through the KEGG (http://www.kegg.jp/) pathway enrichment analysis of significantly differentially expressed proteins, it can help us to understand the metabolic or signal pathways that these proteins may be involved in.

### 2.8 Western Blot Analysis

The 50 mg heart tissue was added to the phenylmethylsulfonyl fluoride (PMSF), phosphatase inhibitor, and RIPA buffer and lysed and cracked for 30 min on the ice to ensure complete cleavage. The protein was quantified with the BCA protein assay kit and then protein was separated by 8% SDS–PAGE. The proteins were transferred to polyvinylidene difluoride (PVDF) membrane and incubated overnight with primary antibodies against PTP1B, IRS-1, P-IRS, HK2, HIF-1 α, and Nrf2, followed by incubation with β-actin and NADPH, which served as an internal control.

### 2.9 Statistical Analysis

The analysis of related data was performed using SPSS software (version 17.0). The experimental data are expressed as the mean ± SD. Data comparison between various experimental groups was performed by T-test or one-way ANOVA. *p < 0.05, **p < 0.01.

## 3 Results

### 3.1 Effect of Doxorubicin on Heart Biochemical Parameters and Pathological Situations of Rat

**FIGURE 1:**
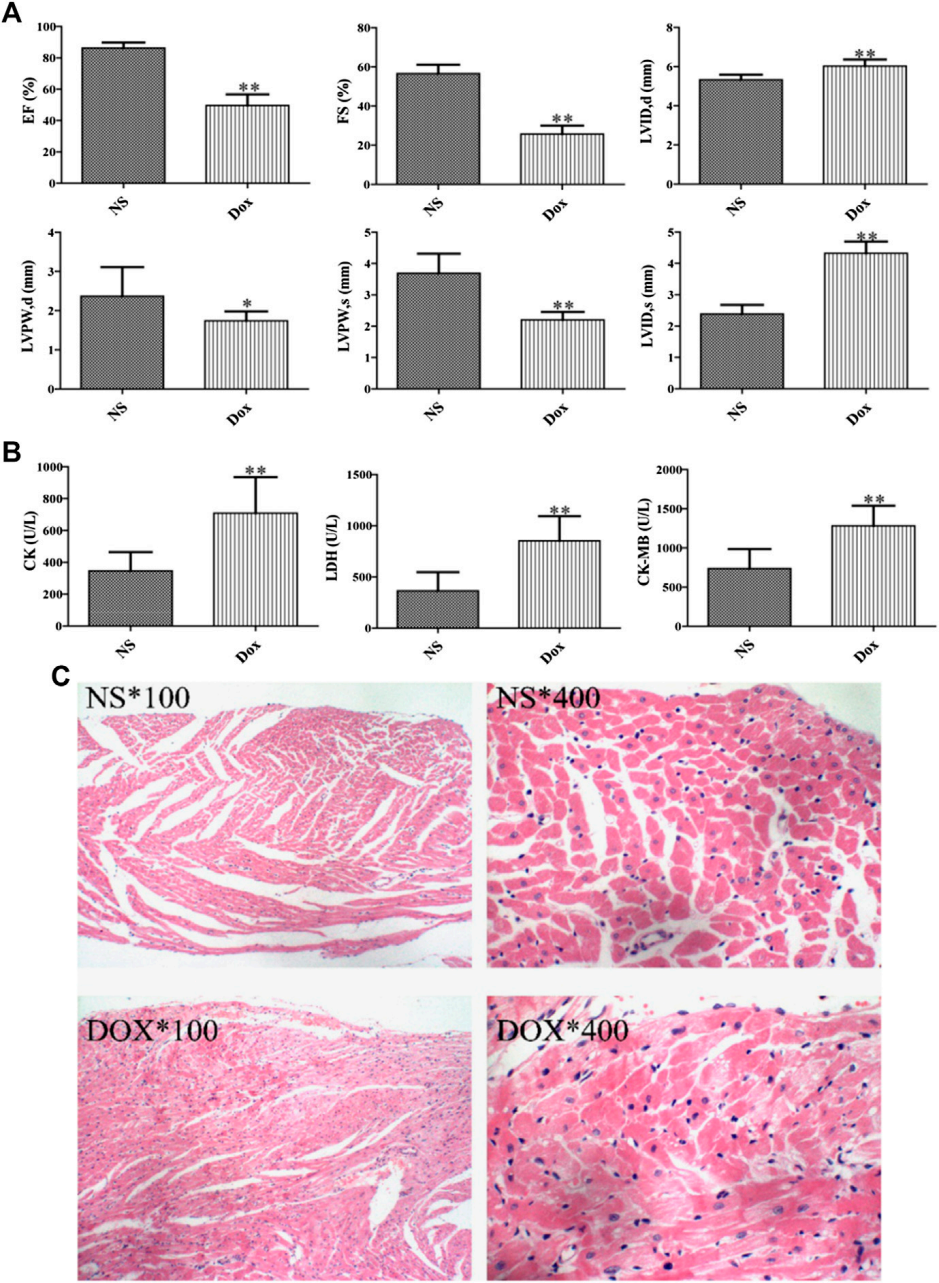
Effects of DOX-induced heart pathology and biochemistry changes in heart failure. **(A)** Echocardiographic evaluation of rat hearts. **(B)** Biochemical indices in rat serum. **(C)** Staining results of rat cardiomyocytes. (*p < 0.05, **p < 0.01. *, 100: magnification ×100; *, 400: magnification ×400).

DOX is an effective cancer chemotherapy agent, which can cause pathological and physiological HF (Fu et al., 2016). Echocardiography directly reflected the ejection capacity, systolic and diastolic function of the heart, and played an irreplaceable role in the diagnosis of cardiotoxicity. We compared the echocardiograms. A significant elevation of serum diastolic left ventricular volume (LVID, d) and systolic left ventricular volume (LVID, s) was observed in DOX group compared with NS group over the entire time course, whereas the levels of EF, left ventricular diameter shortening rate (FS), left ventricular posterior wall systolic thickness (LVPW, s), and left ventricular posterior wall diastolic thickness (LVPW, d) were decreased, as shown in Figure 1A. In addition, we also compared the changes of serum CK, CK–MB, and LDH between the DOX group and the NS group (Figure 1B) and evaluated the degree of cardiotoxicity of DOX in rats. In conclusion, it indicated that the rats presented the typical pathological features of myocardial damage.

The damage severity was further verified by H&E staining of myocardial cell. In Figure 1C, the rat myocardial cells in the NS group are arranged orderly and the texture structure is clear. On the contrary, the myocardial cells in the DOX group had more significant edema, some myocardial nuclei were large and stained deeply, and there were many vacuoles near the nuclei, the obvious infiltration of inflammatory cells can be seen in the stroma, and the myocardial fibers are dissolved and broken. Consequently, combined with the histopathological manifestation results, DOX can affect the normal physiological state of the heart.

### 3.2 Metabolomics Analysis

**FIGURE 2:**
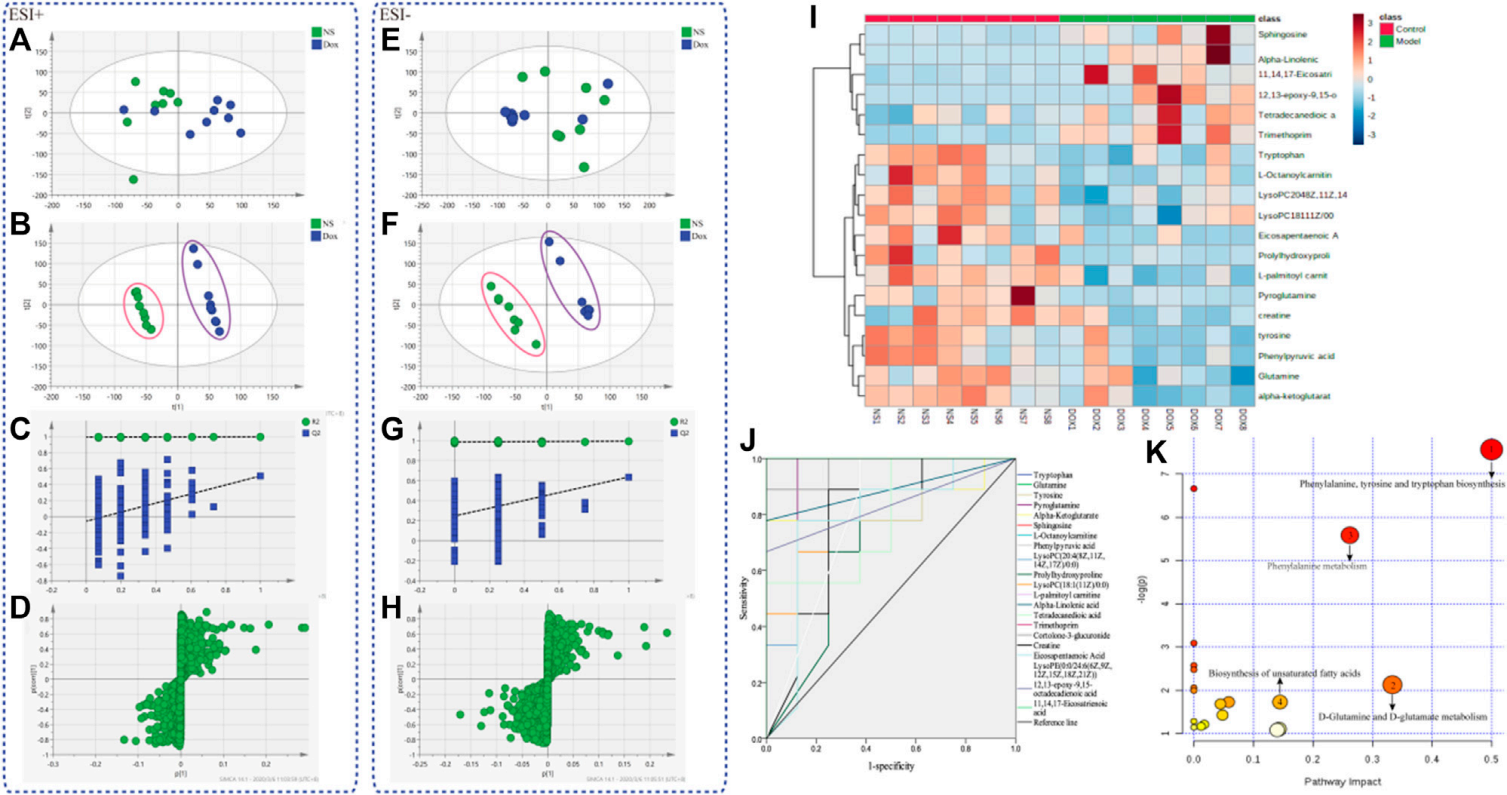
The metabolic profiles and metabolite analysis of plasma samples. **(A)** PCA score plots (ESI+) for comprehensive metabonomic data of plasma samples. Models include the control and DOX-treated rat (3 mg/kg). **(B)** PLS–DA models (ESI+) of UPLC-Q-TOF/MS metabonomics data for NS group and models include the control and DOX-treated rat (3 mg/kg). **(C)** Model validation diagram in positive ion mode. **(D)** S–plot load diagram in positive ion mode. **(E)** PCA score plots (ESI–) for comprehensive metabonomic data of plasma samples. Models include the control and DOX-treated rat (3 mg/kg). **(F)** PLS–DA models (ESI–) of UPLC-Q-TOF/MS metabonomics data for NS group and models include the control and DOX-treated rat (3 mg/kg). **(G)** Model validation diagram in negative ion mode. **(H)** S–plot load diagram in negative ion mode. **(I)** Heat map for metabolites. **(J)** Diagnostic significance of ROC curve analysis for metabolites. **(K)** Metabolic pathway map analysis.

Plasma samples of rats were analyzed by UPLC-Q-TOF/MS to obtain significantly changed metabolites. The original mass spectra data were analyzed with SIMCA software (version 12.0, Sweden) for multivariate statistical analysis respectively. Then PCA (Figures 2A,E) and PLS–DA were calculated (Figures 2B,F). The results showed complete separation of the heart between the NS group and the DOX injury groups in both negative and positive ionization mode, which were described by high values of R2Y (0.996 and 0.996) and Q2 (0.665 and 0.665) parameters. We also performed permutation tests (Figures 2C,G), and the results showed that the PLS–DA model was not overfitted and had high reliability. The model validation diagram is used to diagnose, and the number of tests is set to 200. The obtained model validation diagram proves that the PLS–DA model has no overfitting and high reliability. Meanwhile, S–plot load plot (Figures 2D,H) showed that the farther the metabolites from the central origin on the “S” curve, the greater the VIP value and the greater the contribution. All the differential endogenous metabolites meet the requirements of VIP > 1.5 and *p* < 0.05. By further MS2 identification, 21 significantly changed ions were matched with endogenous metabolites from HMDB database (Table 1); 12 of the identified metabolites were downregulated and nine were upregulated. Next, we will diagnose the 21 differential metabolites’ diagnostic significance by two ways: ROC curve and cluster analysis. The ROC curve (Figure 2J) shows that the area of the 21 metabolites under the curve is all greater than 0.7; the heat map (Figure 2I) results showed that the content changes of 21 biomarkers in the NS group and the DOX group were significantly different, indicating that they had better accuracy in the diagnosis of heart failure induced by DOX. Based on the KEGG pathway enrichment analysis and topological analysis of MetPA database (Figure 2K), we can intuitively see that these differential metabolites participate in a variety of metabolic pathways, among which the metabolic pathways more influential in heart failure include the phenylalanine, tyrosine, and tryptophan biosynthesis, the D-glutamine and D-glutamate metabolism, the phenylalanine metabolism, and the biosynthesis of unsaturated fatty acids. Through these results, we found that HF mainly involves energy metabolism, amino acid metabolism, fatty acid metabolism, and glycerophospholipid metabolism disorders.

**TABLE 1:** Potential biomarkers were selected in plasma of COX-treated rats and normal rats.

No	T_R_ (min)	Compound	Molecular formula	*m/z* obsd	*m/z* calcd	Error (ppm)	Ion mode	Treatment group vs. control group
1	0.93	Tryptophan	C_11_H_12_N_2_O_2_	227.0796	227.0796	0	+	↓**
2	0.92	Tyrosine	C_9_H_11_NO_3_	182.0817	182.0819	1.10	+	↓*
3	0.80	Glutamine	C_5_H_10_N_2_O_3_	169.0589	169.0587	–1.18	+	↓*
4	0.83	Pyroglutamine	C_5_H_8_N_2_O_2_	129.0664	129.0665	0.77	+	↓*
5	11.31	Alpha-ketoglutarate	C_5_H_6_O_5_	184.9852	184.9864	6.49	+	↓**
6	4.41	Sphingosine	C_18_H_37_NO_2_	300.2903	300.2902	–0.33	+	↑*
7	2.80	L-Octanoylcarnitine	C_15_H_29_NO_4_	288.2175	288.2169	–2.08	+	↓*
8	0.93	Phenylpyruvic acid	C_9_H_8_O_3_	165.0552	165.0547	–3.03	+	↑*
9	2.45	Prolylhydroxyproline	C_10_H_16_N_2_O_4_	251.1008	251.1014	2.39	+	↓*
10	5.37	LysoPC(20:4 (8Z,11Z,14Z,17Z)/0:0)	C_28_H_50_NO_7_P	566.3223	566.3218	–0.80	+	↓**
11	6.25	LysoPC(18:1 (11Z)/0:0)	C_26_H_52_NO_7_P	544.3379	544.3381	0.37	+	↑*
12	5.48	L-Palmitoyl carnitine	C_23_H_45_NO_4_	400.3427	400.3432	1.25	+	↓**
13	5.66	Alpha-linolenic acid	C_18_H_30_O_2_	279.2324	279.2314	–3.58	+	↑*
14	6.03	Tetradecanedioic acid	C_14_H_26_O_4_	297.1468	297.15	10.77	+	↑*
15	6.28	Trimethoprim	C_14_H_18_N_4_O_3_	291.1457	291.1437	–6.87	+	↑**
16	5.94	Cortolone-3-glucuronide	C_27_H_42_O_11_	565.2625	565.2661	6.37	+	↓**
17	2.53	Creatine	C4H9N3O2	130.0617	130.0623	4.61	–	↓*
18	5.28	LysoPE (0:0/24:6 (6Z,9Z,12Z,15Z,18Z,21Z))	C_29_H_48_NO_7_P	552.3090	552.3085	–0.91	–	↑*
19	7.69	Eicosapentaenoic acid	C_20_H_30_O_2_	301.2168	301.2188	6.64	–	↓*
20	5.93	12,13-Epoxy-9,15-octadecadienoic acid	C_18_H_30_O_3_	293.2117	293.2119	0.68	–	↑*
21	5.83	11,14,17-Eicosatrienoic acid	C_20_H_34_O_2_	305.2481	305.2500	6.22	–	↑**

The change trend is compared between the DOX group and the NS group. ↑ and ↓ represent up- and downregulation. *p < 0.05. **p < 0.01.

### 3.3 Proteomic Analysis

**FIGURE 3:**
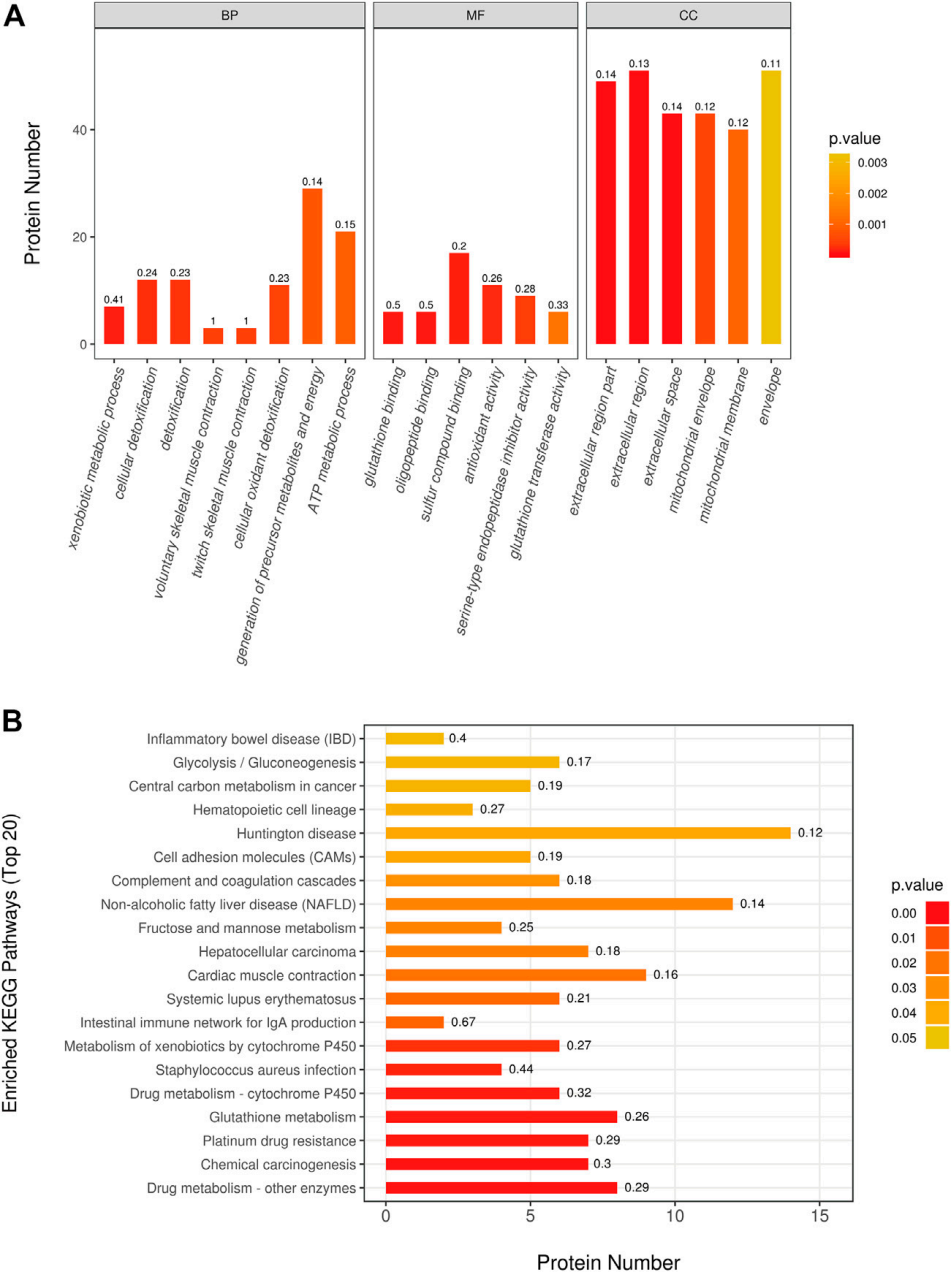
Differentially expressed proteins pathway enrichment analysis. **(A)** The most significantly (*p* < 0.05) enriched GO terms (Top 20) in rat treated with doxorubicin. **(B)** The most significantly (*p* < 0.05) enriched KEGG pathways (Top 20) in rat treated with doxorubicin. (BP: biological process; MF: molecular function; CC: cellular component).

By utilization of TMT-based quantitative proteomics, we identified 29,483 peptides in the rats heart tissue samples of the two groups and identified 3,727 proteins (Supplementary 2), among which 278 proteins were significantly altered (fold change > 1.2 or < 0.83, *p* < 0.05) as a result of doxorubicin treatments. In detail, 118 proteins were upregulated and 160 proteins were downregulated in adriamycin treated group (Supplementary 2). In order to better understand which functions or biological pathways are significantly affected by biological processing, we annotated 278 differential proteins by Blast2Go software (https://www.blast2go.com/) for GO function. It can be seen that significant changes have taken place in the top 20 GO terms (Figure 3A): Biological processes such as the generation of precursor metabolites and ATP metabolic process; molecular functions such as glutathione binding and antioxidant activity; cellular components such as mitochondrial envelope, extracellular space, and extracellular region. Notably, to understand the metabolic or signaling pathways that these proteins may be involved in, we performed KEGG pathway (Figure 3B) annotation and found that significant changes have taken place in important pathways such as glutathione metabolism, drug metabolism–cytochrome P450, cardiac muscle contraction, fructose and mannose metabolism, glycolysis/gluconeogenesis, and complement and coagulation. However, the mechanism is still not clear and needs further clarification.

### 3.4 Integrated Proteomics and Metabolomics Analysis

**FIGURE 4:**
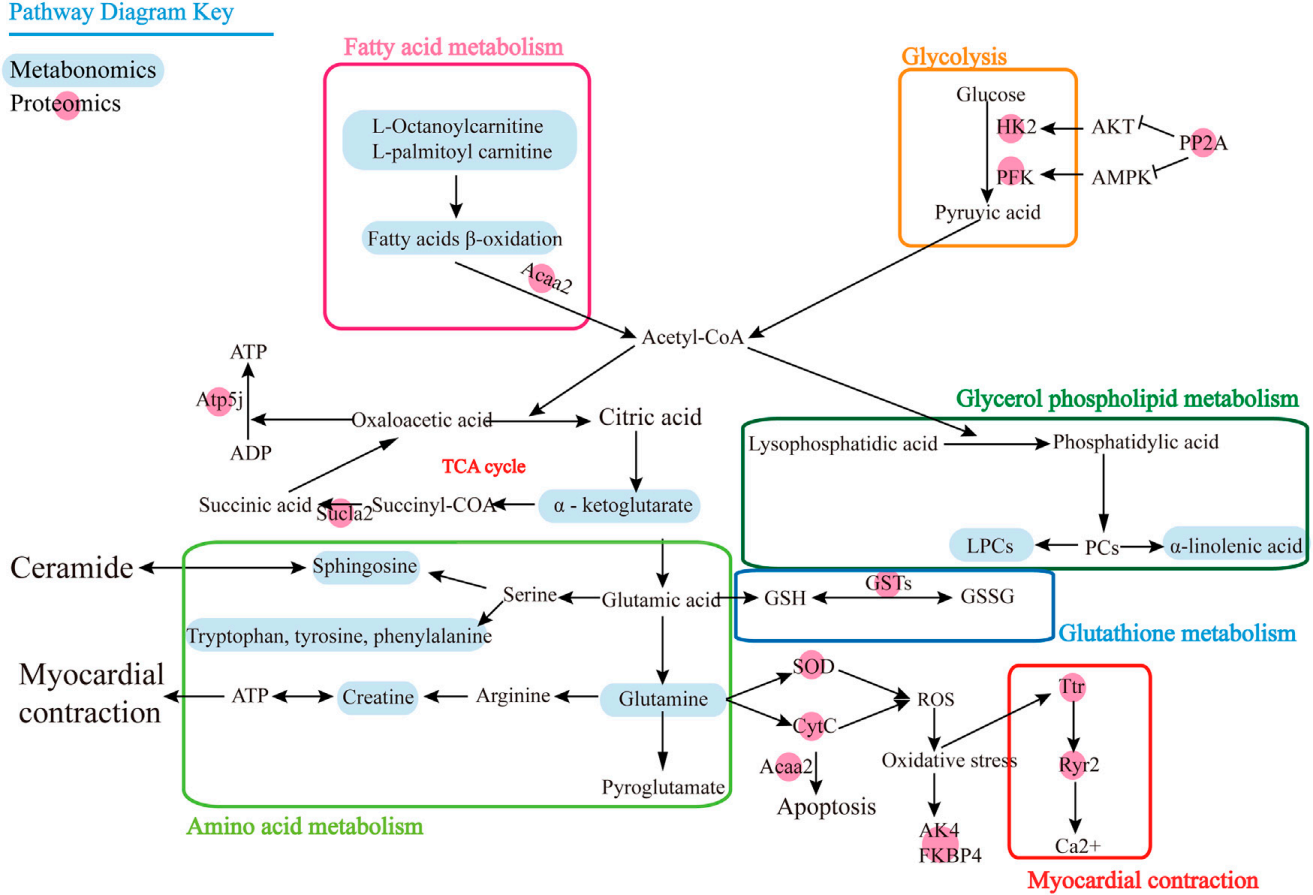
Pathway map for combined analysis of metabolomics and proteomics.

To associate the proteomics data with the metabolomics data, we conducted a joint pathway analysis with differential metabolites and proteins. We found that 25 differential proteins (Table 2) were closely related to the differential metabolites. The results revealed that several metabolic pathways were significantly targeted, including fatty acid metabolism, glycolysis, amino acid metabolism, glycerol phospholipid metabolism, glutathione metabolism, and myocardial contraction (Figure 4), further relating these pathways to the occurrence of heart failure. In fatty acid metabolism, carnitine substances such as L-octanoylcarnitine and L-palmitoylcarnitine and proteins such as acetyl-CoA acyltransferase 2 and acyl-CoA dehydrogenase, C–2 to C–3 short chain affect the decrease of the fatty acid beta-oxidation catalytic enzyme, suggesting that the energy supply process of fatty acid β-oxidation in cardiomyocytes is affected. The upregulation of protein phosphatase and the reduction of glycolytic enzymes in glycolysis indicate that the glycolysis process of cardiomyocytes is also affected. Meanwhile, the decrease of alpha-ketoglutarate, ATP synthase, H+ transporting, mitochondrial Fo complex, subunit F6, and succinate-CoA ligase ADP-forming beta subunit in TCA cycle indicated that the efficiency of TCA cycle was reduced. The decrease of superoxide dismutase 2, mitochondrial, cytochrome c, adenylate kinase 4, and FK506 binding protein 4 suggests the level of oxidative stress was higher. Creatine, transthyretin, and ryanodine receptor 2 decrease indicates that myocardial contraction is affected. The decrease of lysophosphatidylcholine and the increase of alpha-linolenic acid suggested that glycerol phospholipid metabolism has been affected. Elevated expression of glutathione-S-transferase represents the imbalance of glutathione metabolism in cardiomyocytes. We speculate that DOX may lead to inadequate myocardial energy supply and accumulation of cardiac reactive oxygen species through abnormal fatty acid metabolism and glucose metabolism, which together lead to calcium homeostasis affecting cardiac contractile function and lead to HF ultimately.

**TABLE 2:** Differentially regulated proteins selected by integration analysis.

No	Accession	Protein names	Gene name	LP/NS ratio	Regulated type
1	ENSRNOP00000018325	Glutathione S-transferase alpha 1	Gsta1	1.31	↑*
2	ENSRNOP00000010579	Microsomal glutathione S-transferase 1	Mgst1	1.27	↑**
3	ENSRNOP00000024601	Glutathione S-transferase pi 1	Gstp1	1.19	↑**
4	ENSRNOP00000025939	Glutathione S-transferase mu 2	Gstm2	1.17	↑*
5	ENSRNOP00000039050	Glutathione S-transferase mu 1	Gstm1	1.13	↑**
6	ENSRNOP00000075572	Glutathione S-transferase alpha 4	Gsta4	1.10	↑*
7	ENSRNOP00000005612	Enolase 3	Eno3	0.85	↓*
8	ENSRNOP00000018227	Phosphoglycerate mutase 2	Pgam2	0.87	↓*
9	ENSRNOP00000008813	Hexokinase 2	Hk2	0.84	↓**
10	ENSRNOP00000070507	Phosphofructokinase, muscle	Pfkm	0.91	↓*
11	ENSRNOP00000022113	Transthyretin	Ttr	0.61	↓*
12	ENSRNOP00000023601	Ryanodine receptor 2	Ryr2	0.87	↓**
13	ENSRNOP00000060140	Acetyl-CoA acyltransferase 2	Acaa2	0.83	↓**
14	ENSRNOP00000001556	Acyl-CoA dehydrogenase, C–2 to C–3 short chain	Acads	0.86	↓**
15	ENSRNOP00000002116	ATP synthase, H+ transporting, mitochondrial Fo complex, subunit F6	Atp5pf	0.91	↓*
16	ENSRNOP00000060229	Succinate-CoA ligase ADP-forming beta subunit	Sucla2	0.90	↓*
17	ENSRNOP00000025794	Superoxide dismutase 2, mitochondrial	Sod2	0.84	↓*
18	ENSRNOP00000041521	Cytochrome c	Cyt C	0.86	↓*
19	ENSRNOP00000065349	Adenylate kinase 4	AK4	0.91	↓**
20	ENSRNOP00000008737	FK506 binding protein 4	Fkbp4	0.88	↓*
21	ENSRNOP00000068826	COX14, cytochrome c oxidase assembly factor	Cox14	0.85	↓*
22	ENSRNOP00000022487	Cytochrome c oxidase subunit 5B	Cox5b	0.90	↓*
23	ENSRNOP00000025525	Cytochrome c oxidase subunit 5A	Cox5a	0.90	↓**
24	ENSRNOP00000066702	Cytochrome c oxidase subunit 7A1	Cox7a1	0.85	↓*
25	ENSRNOP00000023139	Protein phosphatase 2	PP2A	1.10	↑*

**FIGURE 5:**
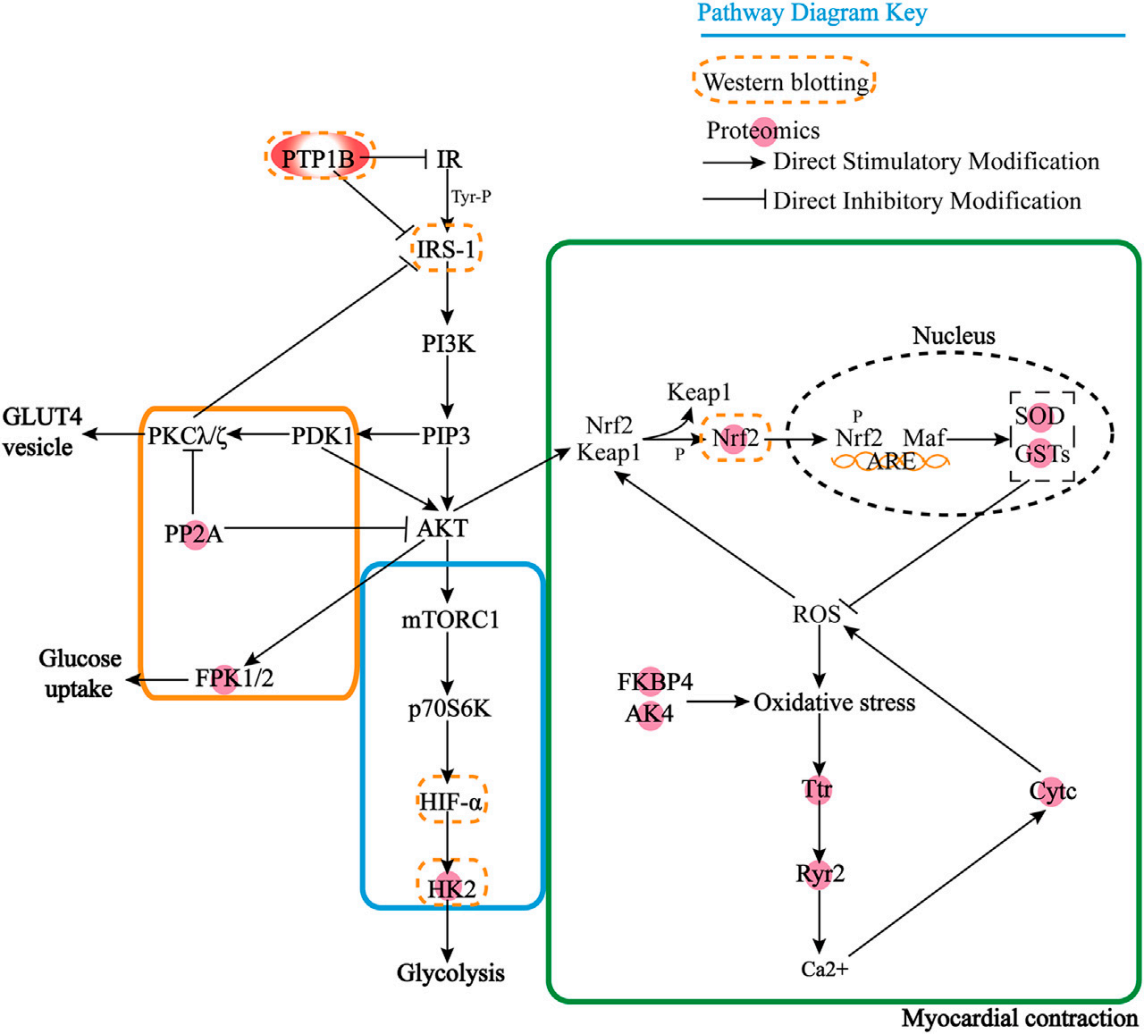
Interaction mechanism between PTP1B and differential proteins.

However, we found that most of these proteins were downstream effector proteins through KEGG analysis, so it is particularly important to find the upstream key target proteins. Then we use the protein prediction function of the STRING database to select the proteins that play an important role in proteomics results to predict the upstream proteins based on the known DOX target proteins, and we enrich the key target protein tyrosine-protein phosphatase non-receptor type 1 (PTP1B). Through the KEGG database, we can find that PTP1B can inhibit insulin receptor (IR) substrate and PI3K achieves GLUT4 transport by activating Akt/PKB and PKC cascade and protein synthesis by mTOR and downstream components after protein kinase B activation. PP2A has an inhibitory effect on Akt and PKC ζ. The negative feedback signal from Akt/PKB, PKC ζ, and p70S6K leads to serine phosphorylation and inactivation of IRS signal transduction, resulting in the formation of HIF-α, and there is a certain correlation between HK2 and HIF-α, which further affects glycolysis. When oxidative stress occurs, protein kinase C and phosphatidylinositol 3-kinase promote the dissociation of Nrf2 and Keap1. Nrf2 enters the nucleus and binds to antioxidant response elements to generate GSTs and SOD, thus exerting the role of antioxidant damage. With the degradation of Nrf2, the activation is terminated. AK4 and FKBP4 also affect oxidative stress, produce Ttr and Ryr2 to release Ca2+, stimulate CytC generation, and at the same time block the scavenging of ROS free radicals and lead to apoptosis. In Figure 5, it can be seen that PTP1B is in the upstream position of the pathway, so we speculate that it is one of the key targets upstream.

### 3.5 Protein Expression in Cardiac Tissue of Two Groups

**FIGURE 6:**
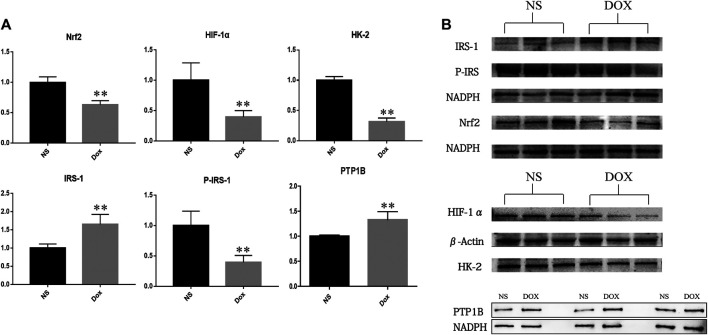
The expression of NS group and DOX group related proteins in heart tissue (*p < 0.05; **p < 0.01). **(A)** Expression level of PTP1B, Nrf2, HIF-1α, HK-2, IRS-1, and P-IRS in NS group and DOX group. **(B)** Protein level of PTP1B, Nrf2, HIF-1α, HK-2, IRS-1, and P-IRS in NS group and DOX group.

Previous studies have shown that PTP1B may be a key upstream target for HF. To verify our hypothesis, we detected the expression of PTP1B and its downstream node proteins IRS, HIF-1 α, Nrf2, and HK-2. In results as shown in Figure 6, we found that, compared with the NS group, the expression level of PTP1B and IRS protein increased significantly, while the expression levels of P-IRS1, HIF-1α, Nrf2, and HK-2 protein decreased significantly in the DOX group. It is suggested that the upregulation of PTP1B protein will inhibit IRS phosphorylation, resulting in decreased P-IRS1 expression, a decrease in the expression of HIF-1α, and inhibition of downstream glycolysis; at the same time, it can also cause decreased expression of Nrf2, leading to increased levels of oxidative stress in the body, promoting the occurrence of HF through energy metabolism and oxidative stress.

## 4 Discussion

Studies have shown that protein PTP1B, a molecular target for anti-type II diabetes, obesity, and cancer treatment, can regulate the level of protein tyrosine phosphorylation in cells (Chen et al., 2018). PTP1B is widely expressed in cardiovascular tissues, especially in the heart and endothelial cells (Thiebaut et al., 2016). Many studies have shown that inhibition of PTP1B can reduce cardiac dysfunction, systemic inflammation, and mortality (Maupoint et al., 2016; Thiebaut et al., 2016). Meanwhile, endothelial cell PTP1B deletion is associated with cardiac vascular endothelial growth factor signaling and angiogenesis and can protect against chronic afterload-induced heart failure (Gogiraju et al., 2016). Therefore, we believe that PTP1B is an interesting molecular target for the treatment of cardiovascular and metabolic diseases. However, the mechanism of PTP1B with the HF is not very clear. In our research, we found that target protein PTP1B can cause damage to the heart through energy metabolism, oxidative stress, and calcium homeostasis disorders.

### 4.1 Energy Metabolism

The heart is the most energy-consuming organ of the body and requires a large amount of ATP to provide energy. Removing mitochondrial oxidative phosphorylation, the main source of ATP is fatty acid oxidation, followed by glucose oxidation, amino acid oxidation, and so on. In heart failure, energy production is converted from fatty acid beta-oxidation to glucose oxidation, which contributes to the progressive deterioration of cardiac function in hypertrophy and heart failure (Dong et al., 2017). In this study, carnitine, Acaa2, and Acads showed a downward trend, which proved that the oxidation ability of fatty acids was affected and cardiac function was impaired (Bertero and Maack, 2018). Studies in knockdown PTP1B mice have shown that PTP1B protein has significant effects on insulin sensitivity, glucose homeostasis, and lipid metabolism (Owen et al., 2013; Zhang et al., 2016). PTP1B attenuates insulin signal transduction by removing tyrosine from activated insulin receptors and IRS-1 triggers PI3K activation and catalyzes the lipid product phosphatidylinositol (3,4,5-) triphosphate, which in turn leads to the activation of protein kinase B to stimulate GLUT4 translocation affecting glucose absorption (Riehle and Abel, 2016; Nguyen et al., 2018; Ormazabal et al., 2018). Meanwhile, PTP1B activates mTOR through the Akt/PI3K pathway, thereby affecting HIF-1α and HK2, and converts to aerobic glycolysis metabolism (Wolf et al., 2011; Cheng et al., 2014).

Pyruvate can be oxidized by acetyl coenzyme A or as a complementary substrate to supplement the intermediate products of the TCA cycle (Turer et al., 2019). Therefore, when the rate-limiting enzyme HK-2, which reacts with glycolytic capacity, and phosphofructokinase (Pfkp, Pfkm), which is a key regulator in glycolysis decrease, lead to a decrease in glucose utilization, pyruvate also decreases (Gibb et al., 2017; Jiao et al., 2018). Meanwhile, the level of nonessential amino acid glutamine also plays a role in regulating cellular energy homeostasis, which is converted into a-ketoglutarate, an intermediate of the citric acid cycle, by a two-step process (Tebay et al., 2015). The decrease of metabolite α-ketoglutaric acid, differential protein atp5j, and sucla2 indicated the decrease of TCA cycle efficiency. This reflects the reduced TCA cycle efficiency of cardiomyocytes and the inability to provide sufficient ATP, indicating that fatty acid oxidation and glycolysis can affect the downstream TCA cycle, resulting in a decrease in ATP.

In the study, we found that the LPCs decreased significantly, which has been demonstrated to be accompanied by a decrease in ejection fraction when cardiovascular disease occurrs, and a decrease in EF is associated with disorders in phospholipid metabolism (Marcinkiewicz-Siemion et al., 2018). Christin Stegemann et al. (Stegemann et al., 2014) analyzed the relationship between molecular lipids and cardiovascular risk and found that LPCs, cholesterol esters, phosphatidylcholine, phosphatidylethanolamine, sphingomyelin, and triacylglycerol were associated with cardiovascular disease. Combined with the above factors, PTP1B protein affected the phosphorylation of insulin receptor substrate protein IRS, caused disorders of glycolysis and lipid metabolism, and was accompanied by the imbalance of glutamine regulatory capacity homeostasis, which together led to the impact of TCA cycle, resulting in the reduction of cardiac energy supply.

### 4.2 Oxidative Stress

Oxidative stress is an important feature of the onset and development of many diseases (including cardiovascular diseases), and one of the common features that play an important role in the pathophysiology of heart failure is chronic oxidative stress (Belch et al., 1991; Hill and Singal, 1997; Zima and Mazurek, 2016). PTP1B increases glutathione S-transferase (GSTs) protein levels by stimulating insulin receptor substrates, activating downstream PI3K, Akt/protein kinase B, ribosomal p70S6 kinase, and PKC (Kim et al., 2006). Gamma 1-glutamyl-1-cysteine glycine (GSH), a tripeptide that can prevent oxidative stress and can be oxidized to form glutathione two sulfur (GSSG), when the ratio between oxidized and reduced GSH increases and glutathione is mixed with disulfide, will play a pathogenic factor in cardiovascular diseases (Lu, 2013; Zima and Mazurek, 2016; Chevallier et al., 2020) The increased expression of glutathione S-transferase represents the imbalance of glutathione metabolism in cardiomyocytes, depletion of GSH increases and the ratio of GSH/GSSG decreases, which enhanced oxidative stress from ROS in cardiomyocytes (Cramer et al., 2017; Ma et al., 2018). Among them, Gstp1 can sensitize cells to free radical-mediated damage by reducing the ability of reactive electrophiles to bind to glutathione, and its main active site is Val105, which is mainly associated with cardiovascular diseases (Doney et al., 2005). The GSTM family is associated with breast cancer, and there is a certain association between breast cancer and oxidative stress (Li et al., 2018). Anthracyclines are also metabolized by GSTMs-mediated reactions, which can accelerate the metabolic inactivation of therapeutic drugs (Tan et al., 2008). GSTA4 is a 2-phase detoxifying enzyme whose expression increases in response to oxidative stress (Shearn et al., 2016). In this study, Gsta1/4, Gstm1/2, and Gstp1GSTs all showed a significant upward trend, demonstrating that doxorubicin-induced heart failure has a strong correlation with oxidative stress.

On the other hand, Sharma Sudha et al. demonstrated that the absence of SOD2 leads to an increase in ROS (Sharma et al., 2020). Nrf2 is widely recognized as a transcription factor activated by oxidative stress, and cells lacking Nrf2 can lead to mitochondrial dysfunction, resulting in increased ROS and impaired antioxidant capacity and further aggravated heart failure (Tebay et al., 2015). Nrf2 plays a crucial role in activating the Sirt3/SOD2 signaling pathway, and studies have demonstrated significant inhibition of silenced Nrf2 cells, SOD2 activity, and GSH/GSSG ratio, as well as increased ROS and MDA levels in induced oxidative stress cell models (Zhou et al., 2019). Abnormal cell membrane potential leads cytochrome C (CytC) to the cytoplasm and enhances apoptosis (Sun Jang et al., 2009; Zhang et al., 2019). And the increase of ROS level will inhibit superoxide dismutase (SOD), catalase (Marwick), GSH-PX activity, and GSH content (Liu et al., 2020). Overexpression of AK4 stabilizes HIF-1a protein by increasing intracellular ROS levels (Jan et al., 2019). In this study, AK4 and HIF-1a showed a downward trend, while Nrf2, SOD, and CytC showed a significant downward trend, indicating that oxidative stress played a crucial role in heart failure. Therefore, GSH, Nrf2, SOD2, and CytC are related to the occurrence of heart failure during oxidative stress.

### 4.3 Calcium Homeostasis

Calcium homeostasis is a central point in maintaining normal cardiac contractility in heart failure. Dysfunction of systolic Ca transport caused by dysfunction of the type 2 ryanodine receptor (RyR2) in the sarcoplasmic reticulum is associated with a variety of heart diseases, including catecholaminergic polymorphic ventricular tachycardia, atrial fibrillation, and HF (Connell et al., 2020). In cardiomyocytes, Ca2+ enters through L-type Ca2+ channels, which mediate the opening of RyR2 channels, allowing Ca2+ to contract from sarcoplasmic reticulum to cytoplasm, thus releasing energy from ATP hydrolysis by binding to enzymes on mitochondria (Kaplan et al., 2003; Gonano and Vila Petroff, 2020). Suetomi et al. studies have shown that when RyR2 deficiency or expression decreases, Ca2+ spontaneously leaks from the sarcoplasmic reticulum (Suetomi et al., 2011), which influences the calcium homeostasis of cardiomyocytes, reduces the production of ATP from mitochondria, and leads to heart failure due to cardiac systolic dysfunction. This study demonstrates that oxidative stress leads to the expression of Ttr protein (Zhang P. et al., 2018), which subsequently affects the low expression of RyR2, leading to Ca 2 + homeostasis disorders affecting myocardial contraction. FK506-binding protein 52 (FKBP52, also known as FKBP4), whose downregulation is associated with cardiomyocyte hypertrophy, regulates Ca2+ signaling in a manner dependent on overexpression of peptidyl-prolyl isomerase (PPIase) (Bandleon et al., 2019).

Also, ATP derived from the conversion of phosphokinase to creatine is an important chemical source of myocardial contraction, and the downregulation of creatine also indicates that cardiac systolic and diastolic function is affected during heart failure (Haris et al., 2014).

## 5 Conclusion

The combined analysis results showed that the occurrence of heart failure was mainly related to the metabolic disorders of fatty acid metabolism, glycolysis, TCA cycle, glycerophospholipid metabolism, glutathione metabolism, and amino acid metabolism. These metabolic pathways are closely related to energy metabolism, oxidative stress, and myocardial contraction. In this study, we focused on the discussion that PTP1B inhibits the expression of hypoxia-inducible factor-1 alpha (HIF-1α) by inhibiting the phosphorylation of IRS, leading to the disorder of fatty acid metabolism and glycolysis; on the other hand, the increased expression of GSTs, which leads to the decrease of GSH/GSSG ratio and the decrease of Nrf2, SOD, Cytc, and AK4 together lead to oxidative stress. Decreased expression of Ryr2 results in abnormal cardiac contraction with abnormal Ca2+ homeostasis and decreased ATP. Heart failure is usually defined as a condition of chronic oxidative stress due to energy shortage, so this study suggests that the occurrence of heart failure may be related to a harmful cycle of calcium homeostasis disorders resulting from increased energy demand and increased workload in HF (Seddon et al., 2007; Zima and Mazurek, 2016). It provides a new strategy for the effective treatment of heart failure, the development of new drugs, and the study of pharmacodynamic material basis, but in this study, only the important target protein PTP1B and the subsequent pathway of heart failure have been preliminarily validated; therefore, further functional validation of the target protein is needed from the cellular and animal levels.

Compared with other omics, data generated by proteomics and metabolomics are more directly related to the pathological symptoms and clinical parameters observed by patients (Rinschen and Saez-Rodriguez, 2020). Proteomics remains the most commonly used tool for discovering new biomarkers (Montaner et al., 2020). At present, the combination of multiomics has become a hot topic in the study of disease mechanism. From a clinical point of view, blood has more easily acquired advantages and plasma/serum metabolomics integrated systemic metabolism has better diagnostic and prognostic value than conventional biomarkers (Cheng et al., 2015; Hunter et al., 2016; McGarrah et al., 2018). In this study, we found the changes of metabolites and proteins during heart failure in rats by metabolomics and proteomics, explained the mechanism of heart failure at the phenotypic and genotypic levels, and provided a reference for the study of DOX-induced heart failure. However, in order to provide more information related to the heart’s own metabolism, we need further studies of tissue metabolomics to reflect molecular processes closer to the disease state.

## Data Availability Statement

The original contributions presented in the study are included in the article/Supplementary Material, further inquiries can be directed to the corresponding authors.

## Ethics Statement

The animal study was reviewed and approved by the Institutional Animal Care and Use Committee of Tianjin University of Traditional Chinese Medicine (IACUC), and was conducted in accordance to the guidelines of the National Institutes of Health Animal Care and Use Committee.

## Author Contributions

YW and YL planned and supervised the experiments. SF, WH, YY, YW, and LS analyzed and interpretated the data. YY and YW wrote the article. All authors agreed to the publication. The authors read and approved the final manuscript.

## Funding

This work was supported by the Tianjin Development Program for Innovation and Entrepreneurship and National Natural Science Foundation of China (No. 81903938).

## Conflict of Interest

The authors declare that the research was conducted in the absence of any commercial or financial relationships that could be construed as a potential conflict of interest.

## References

[B1] Bandleon S. Strunz P. P. Pickel S. Tiapko O. Cellini A. Miranda-Laferte E. (2019). FKBP52 regulates TRPC3-dependent Ca^2+^ signals and the hypertrophic growth of cardiomyocyte cultures. J. Cell Sci. 132, 231506 10.1242/jcs.231506 31540954

[B2] Belch J. J. Bridges A. B. Scott N. Chopra M. (1991). Oxygen free radicals and congestive heart failure. Heart 65, 245–248. 10.1136/hrt.65.5.245 PMC1024624 2039668

[B3] Bertero E. Maack C. (2018). Metabolic remodelling in heart failure. Nat. Rev. Cardiol. 15, 457–470. 10.1038/s41569-018-0044-6 29915254

[B4] Chen X. Gan Q. Feng C. Liu X. Zhang Q. (2018). Virtual screening of novel and selective inhibitors of protein tyrosine phosphatase 1B over T-cell protein tyrosine phosphatase using a bidentate inhibition strategy. J. Chem. Inf. Model. 58, 837–847. 10.1021/acs.jcim.8b00040 29608303

[B5] Cheng M.-L. Wang C.-H. Shiao M.-S. Liu M.-H. Huang Y.-Y. Huang C.-Y. (2015). Metabolic disturbances identified in plasma are associated with outcomes in patients with heart failure. J. Am. Coll. Cardiol. 65, 1509–1520. 10.1016/j.jacc.2015.02.018 25881932

[B6] Cheng S.-C. Quintin J. Cramer R. A. Shepardson K. M. Saeed S. Kumar V. (2014). mTOR- and HIF-1 -mediated aerobic glycolysis as metabolic basis for trained immunity. Science 345, 1250684 10.1126/science.1250684 25258083 PMC4226238

[B7] Chevallier V. Andersen M. R. Malphettes L. (2020). Oxidative stress‐alleviating strategies to improve recombinant protein production in CHO cells. Biotechnol. Bioeng. 117, 1172–1186. 10.1002/bit.27247 31814104 PMC7078918

[B8] Connell P. Word T. A. Wehrens X. H. T. (2020). Targeting pathological leak of ryanodine receptors: preclinical progress and the potential impact on treatments for cardiac arrhythmias and heart failure. Expert Opin. Ther. Targets 24, 25–36. 10.1080/14728222.2020.1708326 31869254 PMC6956596

[B9] Cramer S. L. Saha A. Liu J. Tadi S. Tiziani S. Yan W. (2017). Systemic depletion of L-cyst(e)ine with cyst(e)inase increases reactive oxygen species and suppresses tumor growth. Nat. Med. 23, 120–127. 10.1038/nm.4232 27869804 PMC5218918

[B10] Doney A. S. F. Lee S. Leese G. P. Morris A. D. Palmer C. N. A. (2005). Increased cardiovascular morbidity and mortality in type 2 diabetes is associated with the glutathione S transferase theta-null genotype. Circulation 111, 2927–2934. 10.1161/circulationaha.104.509224 15927971

[B11] Dong Z. Zhao P. Xu M. Zhang C. Guo W. Chen H. (2017). Astragaloside IV alleviates heart failure via activating PPARα to switch glycolysis to fatty acid β-oxidation. Sci. Rep. 7, 2691 10.1038/s41598-017-02360-5 28578382 PMC5457407

[B12] Eisenberg E. Di Palo K. E. Piña I. L., (2018). Sex differences in heart failure. Clin. Cardiol. 41, 211–216. 10.1002/clc.22917 29485677 PMC6489832

[B13] Feijen E. A. M. Leisenring W. M. Stratton K. L. Ness K. K. van der Pal H. J. H. van Dalen E. C. (2019). Derivation of anthracycline and anthraquinone equivalence ratios to doxorubicin for late-onset cardiotoxicity. JAMA Oncol. 5, 864–871. 10.1001/jamaoncol.2018.6634 30703192 PMC6490232

[B14] Fu H. Y. Sanada S. Matsuzaki T. Liao Y. Okuda K. Yamato M. (2016). Chemical endoplasmic reticulum chaperone alleviates doxorubicin-induced cardiac dysfunction. Circ. Res. 118, 798–809. 10.1161/circresaha.115.307604 26838784

[B15] Galán-Arriola C. Lobo M. Vílchez-Tschischke P. J. López G. J. de Molina-Iracheta A. Pérez-Martínez C. (2019). Serial magnetic resonance imaging to identify early stages of anthracycline-induced cardiotoxicity. J. Am. Coll. Cardiol. 73, 779–791. 10.1016/j.jacc.2018.11.046 30784671

[B16] Gibb A. A. Lorkiewicz P. K. Zheng Y.-T. Zhang X. Bhatnagar A. Jones S. P. (2017). Integration of flux measurements to resolve changes in anabolic and catabolic metabolism in cardiac myocytes. Biochem. J. 474, 2785–2801. 10.1042/bcj20170474 28706006 PMC5545928

[B17] Gogiraju R. Schroeter M. R. Bochenek M. L. Hubert A. Münzel T. Hasenfuss G. (2016). Endothelial deletion of protein tyrosine phosphatase-1B protects against pressure overload-induced heart failure in mice. Cardiovasc. Res. 111, 204–216. 10.1093/cvr/cvw101 27207947

[B18] Gonano L. A. Vila Petroff M. (2020). Direct modulation of RyR2 leading to a TRICky Ca 2+ balance. Circ. Res. 126, 436–438. 10.1161/circresaha.120.316532 32078455

[B19] Haris M. Singh A. Cai K. Kogan F. McGarvey J. DeBrosse C. (2014). A technique for *in vivo* mapping of myocardial creatine kinase metabolism. Nat. Med. 20, 209–214. 10.1038/nm.3436 24412924 PMC4127628

[B20] Hill M. F. Singal P. K. (1997). Right and left myocardial antioxidant responses during heart failure subsequent to myocardial infarction. Circulation 96, 2414–2420. 10.1161/01.cir.96.7.2414 9337218

[B21] Hoffman J. Lyu Y. Pletcher S. D. Promislow D. E. L. (2017). Proteomics and metabolomics in ageing research: from biomarkers to systems biology. Essays Biochem. 61, 379–388. 10.1042/EBC20160083 28698311 PMC5743054

[B22] Hunter W. G. Kelly J. P. McGarrah R. W. Kraus W. E. Shah S. H. (2016). Metabolic dysfunction in heart failure: diagnostic, prognostic, and pathophysiologic insights from metabolomic profiling. Curr. Heart Fail. Rep. 13, 119–131. 10.1007/s11897-016-0289-5 27216948 PMC5504685

[B23] Jan Y. Lai T. Yang C. Lin Y. Huang M. Hsiao M. (2019). Adenylate kinase 4 modulates oxidative stress and stabilizes HIF-1α to drive lung adenocarcinoma metastasis. J. Hematol. Oncol. 12, 12 10.1186/s13045-019-0698-5 30696468 PMC6352453

[B24] Jiao L. Zhang H.-L. Li D.-D. Yang K.-L. Tang J. Li X. (2018). Regulation of glycolytic metabolism by autophagy in liver cancer involves selective autophagic degradation of HK2 (hexokinase 2). Autophagy 14, 671–684. 10.1080/15548627.2017.1381804 28980855 PMC5959330

[B25] Kaplan P. Babusikova E. Lehotsky J. Dobrota D. (2003). Free radical-induced protein modification and inhibition of Ca2+-ATPase of cardiac sarcoplasmic reticulum. Mol. Cell Biochem. 248, 41–47. 10.1023/a:1024145212616 12870653

[B26] Kim S. K. Abdelmegeed M. A. Novak R. F. (2006). Identification of the insulin signaling cascade in the regulation of alpha-class glutathione S-transferase expression in primary cultured rat hepatocytes. J. Pharmacol. Exp. Therapeut. 316, 1255–1261. 10.1124/jpet.105.096065 16293713

[B27] Li S. Lang G. T. Zhang Y. Z. Yu K. D. Shao Z. M. Zhang Q. (2018). Interaction between glutathione S‐transferase M1‐null/present polymorphism and adjuvant chemotherapy influences the survival of breast cancer. Cancer Med. 7, 4202–4207. 10.1002/cam4.1567 30032483 PMC6143941

[B28] Liu Q. Wang W. Zhang Y. Cui Y. Xu S. Li S. (2020). Bisphenol A regulates cytochrome P450 1B1 through miR-27b-3p and induces carp lymphocyte oxidative stress leading to apoptosis. Fish Shellfish Immunol. 102, 489–498. 10.1016/j.fsi.2020.05.009 32430284

[B29] Lu S. C. (2013). Glutathione synthesis. Biochim. Biophys. Acta Gen. Subj. 1830, 3143–3153. 10.1016/j.bbagen.2012.09.008 PMC3549305 22995213

[B30] Ma R. Ji T. Zhang H. Dong W. Chen X. Xu P. (2018). A Pck1-directed glycogen metabolic program regulates formation and maintenance of memory CD8+ T cells. Nat. Cell Biol. 20, 21–27. 10.1038/s41556-017-0002-2 29230018

[B31] Marcinkiewicz-Siemion M. Ciborowski M. Ptaszynska-Kopczynska K. Szpakowicz A. Lisowska A. Jasiewicz M. (2018). LC-MS-based serum fingerprinting reveals significant dysregulation of phospholipids in chronic heart failure. J. Pharmaceut. Biomed. Anal. 154, 354–363. 10.1016/j.jpba.2018.03.027 29571133

[B32] Marwick T. H. (2015). The role of echocardiography in heart failure. J. Nucl. Med. 56, 31–38. 10.2967/jnumed.114.150433 26033901

[B33] Mato J. M. Martínez-Chantar M. L. Lu S. C. (2014). Systems biology for hepatologists. Hepatology 60, 736–743. 10.1002/hep.27023 24449428 PMC4105331

[B34] Maupoint J. Besnier M. Gomez E. Bouhzam N. Henry J.-P. Boyer O. (2016). Selective vascular endothelial protection reduces cardiac dysfunction in chronic heart failure. Circ. Heart Fail. 9, e002895 10.1161/circheartfailure.115.002895 27059805

[B35] Maurer M. Packer M. (2020). How should physicians assess myocardial contraction?: redefining heart failure with a preserved ejection fraction. JACC Cardiovasc. Imaging 13, 873–878. 10.1016/j.jcmg.2019.12.021 32139035

[B36] McGarrah R. W. Crown S. B. Zhang G.-F. Shah S. H. Newgard C. B. (2018). Cardiovascular metabolomics. Circ. Res. 122, 1238–1258. 10.1161/circresaha.117.311002 29700070 PMC6029726

[B37] Montaner J. Ramiro L. Simats A. Tiedt S. Makris K. Jickling G. C. (2020). Multilevel omics for the discovery of biomarkers and therapeutic targets for stroke. Nat. Rev. Neurol. 16, 247–264. 10.1038/s41582-020-0350-6 32322099

[B38] Nguyen T. Schwarzer M. Schrepper A. Amorim P. A. Blum D. Hain C. (2018). Increased protein tyrosine phosphatase 1B (PTP1B) activity and cardiac insulin resistance precede mitochondrial and contractile dysfunction in pressure-overloaded hearts. J. Am. Heart Assoc. 7, e008865 10.1161/JAHA.118.008865 29929988 PMC6064925

[B39] Ormazabal P. Scazzocchio B. Varì R. Santangelo C. D’Archivio M. Silecchia G. (2018). Effect of protocatechuic acid on insulin responsiveness and inflammation in visceral adipose tissue from obese individuals: possible role for PTP1B. Int. J. Obes. 42, 2012–2021. 10.1038/s41366-018-0075-4 29769704

[B40] Owen C. Lees E. K. Grant L. Zimmer D. J. Mody N. Bence K. K. (2013). Inducible liver-specific knockdown of protein tyrosine phosphatase 1B improves glucose and lipid homeostasis in adult mice. Diabetologia 56, 2286–2296. 10.1007/s00125-013-2992-z 23832083

[B41] Riehle C. Abel E. D. (2016). Insulin signaling and heart failure. Circ. Res. 118, 1151–1169. 10.1161/circresaha.116.306206 27034277 PMC4833475

[B42] Rinschen M. Saez-Rodriguez J. (2020). The tissue proteome in the multi-omic landscape of kidney disease. Nat. Rev. Nephrol. 10.1038/s41581-020-00348-5 33028957

[B43] Rochette L. Guenancia C. Gudjoncik A. Hachet O. Zeller M. Cottin Y. (2015). Anthracyclines/trastuzumab: new aspects of cardiotoxicity and molecular mechanisms. Trends Pharmacol. Sci. 36, 326–348. 10.1016/j.tips.2015.03.005 25895646

[B44] Russo M. Guida F. Paparo L. Trinchese G. Aitoro R. Avagliano C. (2019). The novel butyrate derivative phenylalanine‐butyramide protects from doxorubicin‐induced cardiotoxicity. Eur. J. Heart Fail. 21, 519–528. 10.1002/ejhf.1439 30843309

[B45] Seddon M. Looi Y. H. Shah A. M. (2007). Oxidative stress and redox signalling in cardiac hypertrophy and heart failure. Heart 93, 903–907. 10.1136/hrt.2005.068270 16670100 PMC1994416

[B46] Sharma S. Bhattarai S. Ara H. Sun G. St Clair D. K. Bhuiyan M. S. (2020). SOD2 deficiency in cardiomyocytes defines defective mitochondrial bioenergetics as a cause of lethal dilated cardiomyopathy. Redox Biol. 37, 101740 10.1016/j.redox.2020.101740 33049519 PMC7559509

[B47] Shearn C. T. Fritz K. S. Shearn A. H. Saba L. M. Mercer K. E. Engi B. (2016). Deletion of GSTA4-4 results in increased mitochondrial post-translational modification of proteins by reactive aldehydes following chronic ethanol consumption in mice. Redox Biol. 7, 68–77. 10.1016/j.redox.2015.11.013 26654979 PMC4683459

[B48] Stegemann C. Pechlaner R. Willeit P. Langley S. R. Mangino M. Mayr U. (2014). Lipidomics profiling and risk of cardiovascular disease in the prospective population-based Bruneck study. Circulation 129, 1821–1831. 10.1161/circulationaha.113.002500 24622385

[B49] Suetomi T. Yano M. Uchinoumi H. Fukuda M. Hino A. Ono M. (2011). Mutation-linked defective interdomain interactions within ryanodine receptor cause aberrant Ca 2+ release leading to catecholaminergic polymorphic ventricular tachycardia. Circulation 124, 682–694. 10.1161/circulationaha.111.023259 21768539 PMC3153588

[B50] Sun Jang J. Piao S. Cha Y.-N. Kim C. (2009). Taurine chloramine activates Nrf2, increases HO-1 expression and protects cells from death caused by hydrogen peroxide. J. Clin. Biochem. Nutr. 45, 37–43. 10.3164/jcbn.08-262 19590705 PMC2704325

[B51] Tan S. Lee S.-C. Goh B.-C. Wong J. (2008). Pharmacogenetics in breast cancer therapy. Clin. Cancer Res. 14, 8027–8041. 10.1158/1078-0432.CCR-08-0993 19088019

[B52] Tebay L. E. Robertson H. Durant S. T. Vitale S. R. Penning T. M. Dinkova-Kostova A. T. (2015). Mechanisms of activation of the transcription factor Nrf2 by redox stressors, nutrient cues, and energy status and the pathways through which it attenuates degenerative disease. Free Radic. Biol. Med. 88, 108–146. 10.1016/j.freeradbiomed.2015.06.021 26122708 PMC4659505

[B53] Thiebaut P.-A. Besnier M. Gomez E. Richard V. (2016). Role of protein tyrosine phosphatase 1B in cardiovascular diseases. J. Mol. Cell. Cardiol. 101, 50–57. 10.1016/j.yjmcc.2016.09.002 27596049

[B54] Turer A. Altamirano F. Schiattarella G. G. May H. Gillette T. G. Malloy C. R. (2019). Remodeling of substrate consumption in the murine sTAC model of heart failure. J. Mol. Cell. Cardiol. 134, 144–153. 10.1016/j.yjmcc.2019.07.007 31340162 PMC6704481

[B55] Wolf A. Agnihotri S. Micallef J. Mukherjee J. Sabha N. Cairns R. (2011). Hexokinase 2 is a key mediator of aerobic glycolysis and promotes tumor growth in human glioblastoma multiforme. J. Exp. Med. 208, 313–326. 10.1084/jem.20101470 21242296 PMC3039857

[B56] Yuan Y. Fan S. Shu L. Huang W. Xie L. Bi C. (2020). Exploration the mechanism of doxorubicin-induced heart failure in rats by integration of proteomics and metabolomics data. Res. Square, 1–24. 10.21203/rs.3.rs-58887/v1 PMC7758990 33362553

[B57] Zhang H. Xu A. Sun X. Yang Y. Zhang L. Bai H. (2020). Self-maintenance of cardiac resident reparative macrophages attenuates doxorubicin-induced cardiomyopathy through the SR-A1-C-MYC axis. Circ. Res. 127, 610–627. 10.1161/circresaha.119.316428 32466726

[B58] Zhang K. W. Finkelman B. S. Gulati G. Narayan H. K. Upshaw J. Narayan V. (2018). Abnormalities in 3-dimensional left ventricular mechanics with anthracycline chemotherapy are associated with systolic and diastolic dysfunction. J. Am. Coll. Cardiol. 11, 1059–1068. 10.1016/j.jcmg.2018.01.015 PMC6417916 29550306

[B59] Zhang P. Guan X. Yang M. Zeng L. Liu C. (2018). Roles and potential mechanisms of selenium in countering thyrotoxicity of DEHP. Sci. Total Environ. 619-620, 732–739. 10.1016/j.scitotenv.2017.11.169 29161598

[B60] Zhang X. Tian J. Li J. Huang L. Wu S. Liang W. (2016). A novel protein tyrosine phosphatase 1B inhibitor with therapeutic potential for insulin resistance. Br. J. Pharmacol. 173, 1939–1949. 10.1111/bph.13483 26990621 PMC4882499

[B61] Zhang Y. Wang X. Chen C. An J. Shang Y. Li H. (2019). Regulation of TBBPA-induced oxidative stress on mitochondrial apoptosis in L02 cells through the Nrf2 signaling pathway. Chemosphere 226, 463–471. 10.1016/j.chemosphere.2019.03.167 30951941

[B62] Zhou Q. Wang X. Shao X. Wang H. Liu X. Ke X. (2019). Tert-butylhydroquinone treatment alleviates contrast-induced nephropathy in rats by activating the Nrf2/Sirt3/SOD2 signaling pathway. Oxid. Med. Cell Longev. 2019, 4657651 10.1155/2019/4657651 31929854 PMC6939416

[B63] Zima A. V. Mazurek S. R. (2016). Functional impact of ryanodine receptor oxidation on intracellular calcium regulation in the heart. Rev. Physiol. Biochem. Pharmacol. 171, 39–62. 10.1007/112_2016_2 27251471 PMC5033687

