# Supplementary material for: Exploration the Mechanism of Doxorubicin-Induced Heart Failure in Rats by Integration of Proteomics and Metabolomics Data
Source: Front Pharmacol. 2020 Dec 10;11:600561. doi: 10.3389/fphar.2020.600561 (PMC7758990; doi:10.3389/fphar.2020.600561)
Supplement: Supplementary file 1 [file datasheet1.docx]

Supplementary 1

**1.Protein Extraction and Peptide Segment Enzymolysis**

Heart tissue was taken and added with appropriate amount of SDT lysate, transferred to a 2 ml centrifuge tube pre-loaded with appropriate amount of quartz sand and a 1/4 Inch Ceramic bead, and homogenized by MP homogenizer (24 x 2, 6.0 M/S, 60 s, twice), followed by ultrasound (80 W, 10 s work, 15 s intermittent, 10 cycles).Boiling water bath for 15 min.Subsequently, centrifugation was performed to remove remaining debris at 12,000 g (4 °C) for 40 min and filtered with 0.22µm filter membrane to collect the filtrate. The protein concentration in the supernatant was determined with BCA kit (Beyotime, China).

**2、SDS-PAGE Separation**

20 µg of proteins for each sample were mixed with 5X loading buffer respectively and boiled for 5 min. The proteins were separated on 12.5% SDS-PAGE gel (constant current 14 mA, 90 min). Protein bands were visualized by Coomassie Blue R-250 staining.


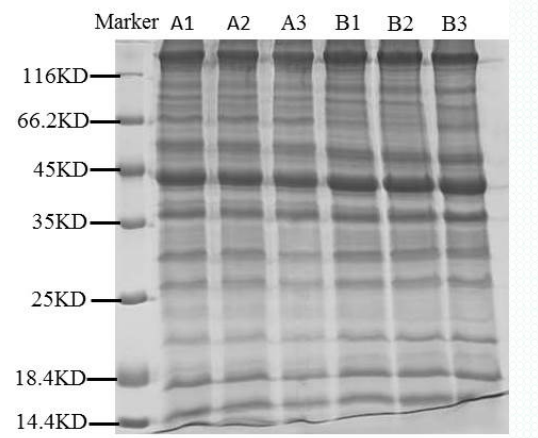


**3、FASP enzymatic hydrolysis**

200 μg of proteins for each sample were incorporated into 30 μl SDT buffer (4% SDS,100 mM DTT, 150 mM Tris-HCl pH 8.0). The detergent, DTT and other low-molecular-weight components were removed using UA buffer (8 M Urea, 150 mM Tris-HCl pH 8.0) by repeated ultrafiltration(Microcon units, 10 kD). Then 100 μl iodoacetamide (100 mM IAA in UA buffer) was added to block reduced cysteine residues and the samples were incubated for 30 min in darkness. The filters were washed with100 μl UA buffer three times and then 100 μl 100 mM TEAB buffer twice. Finally, the protein suspensions were digested with 4 μg trypsin (Promega) in 40 μl TEAB buffer overnight at 37 °C, and the resulting peptides were collected as a filtrate. The peptide content was estimated by UV light spectral density at 280 nm using an extinctions coefficient of 1.1 of 0.1% (g/l) solution that was calculated on the basis of the frequency of tryptophan and tyrosine in vertebrate proteins.

**4、TMT Labeling**

100 μg peptide mixture of each sample was labeled using TMT reagent [7] according to the manufacturer’s instructions (Thermo Fisher Scientific).

**5、Peptide Fractionation with High pH reversed-phase**

The labeled peptides in each group were mixed in the same amount and graded by HighpHReversed-PhasePeptideFractionationKit. Firstly, acetonitrile and 0.1% trifluoroacetic acid (TFA) were used for column equilibrium, and then the mixed labeled peptide samples were added to pure water and desalted at low speed by centrifugation. Finally, the column-bound peptides were gradient eluted with high pH acetonitrile solution with increased concentration in turn. After vacuum drying of each eluted peptide sample, the freeze-dried sample was redissolved with 12 μL 0.1% FA, and the peptide concentration was determined by OD280.

**6、Data Analysis**

MS/MS spectra were searched using MASCOT engine (Matrix Science, London, UK; version 2.2) embedded into Proteome Discoverer 1.4. The following parameters were set.

| Item | Value |
| --- | --- |
| Enzyme | Trypsin |
| Max Missed Cleavages | 2 |
| Fixed modifications | Carbamidomethyl (C),  TMT 6/10plex (N-term),TMT6/10 plex (K) |
| Variable modifications | Oxidation (M) , TMT 6/10plex (Y) |
| Peptide Mass Tolerance | ± 20 ppm |
| Fragment Mass Tolerance | 0.1Da |
| Database | Uniport Database, non-redundant Database, Gene Ontology Database ,KEGG Database ,STRING Database. |
| Database pattern | Decoy |
| Protein Quantification | The protein ratios are calculated as the median of only unique peptides of the protein |
| Experimental Bias | Normalizes all peptide ratios by the median protein ratio. The median protein ratio should be 1 after the normalization. |
